# Automatic classification of communication logs into implementation stages via text analysis

**DOI:** 10.1186/s13012-016-0483-6

**Published:** 2016-09-06

**Authors:** Dingding Wang, Mitsunori Ogihara, Carlos Gallo, Juan A. Villamar, Justin D. Smith, Wouter Vermeer, Gracelyn Cruden, Nanette Benbow, C. Hendricks Brown

**Affiliations:** 1 Department of Computer Science, Florida Atlantic University, 777 Glades Road EE 403, Boca Raton, FL USA; 2 Department of Computer Science and Center for Computational Science, University of Miami, 1320 S. Dixie Highway, Miami, FL USA; 3 Center for Prevention Implementation Methodology, Department of Psychiatry and Behavioral Sciences, Feinberg School of Medicine Northwestern University, 750 N. Lake Shore Dr., Chicago, IL USA; 4 Department of Health Policy and Management, University of North Carolina, Chapel Hill, 135 Dauer Drive, Chapel Hill, NC USA

**Keywords:** Machine learning, Unobtrusive measures, Text mining, Social systems informatics

## Abstract

**Background:**

To improve the quality, quantity, and speed of implementation, careful monitoring of the implementation process is required. However, some health organizations have such limited capacity to collect, organize, and synthesize information relevant to its decision to implement an evidence-based program, the preparation steps necessary for successful program adoption, the fidelity of program delivery, and the sustainment of this program over time. When a large health system implements an evidence-based program across multiple sites, a trained intermediary or broker may provide such monitoring and feedback, but this task is labor intensive and not easily scaled up for large numbers of sites.

We present a novel approach to producing an automated system of monitoring implementation stage entrances and exits based on a computational analysis of communication log notes generated by implementation brokers. Potentially discriminating keywords are identified using the definitions of the stages and experts’ coding of a portion of the log notes. A machine learning algorithm produces a decision rule to classify remaining, unclassified log notes.

**Results:**

We applied this procedure to log notes in the implementation trial of multidimensional treatment foster care in the California 40-county implementation trial (CAL-40) project, using the stages of implementation completion (SIC) measure. We found that a semi-supervised non-negative matrix factorization method accurately identified most stage transitions. Another computational model was built for determining the start and the end of each stage.

**Conclusions:**

This automated system demonstrated feasibility in this proof of concept challenge. We provide suggestions on how such a system can be used to improve the speed, quality, quantity, and sustainment of implementation. The innovative methods presented here are not intended to replace the expertise and judgement of an expert rater already in place. Rather, these can be used when human monitoring and feedback is too expensive to use or maintain. These methods rely on digitized text that already exists or can be collected with minimal to no intrusiveness and can signal when additional attention or remediation is required during implementation. Thus, resources can be allocated according to need rather than universally applied, or worse, not applied at all due to their cost.

## Background

A critical challenge to implementation science is the development of methods to support the scale up of evidence-based programs that are both effective and feasible for use in research-limited community service agencies. Since implementation agencies at the federal, state, or community level typically operate with limited resources, allocating them wisely to achieve a saturated, effective level of implementation (e.g., maximizing the number of adopters and ensuring that each adopter executes the intervention program at a high level of fidelity) is a key issue. A community service agency, local health organization, or technical assistance center may need to take an additional step to move a stagnating but promising implementation forward or may have to withdraw resources from an effort that shows no sign of progressing or achieving impact. Currently, agencies make strategic resource allocation decisions based upon their personal experiences or on limited prior evidence, which is typically derived from investigations conducted under conditions that may not be comparable to real-world circumstances (e.g., implementation trials taking place in a different health system). Ideally, one would want to respond to experiences in the field based on valid and reliable measures to assess the quality, quantity, speed, and sustainment of implementation of an evidence-based intervention through the various stages in the process [3, 7, 29]. However, the accurate and reliable measurement of implementation stages and milestone attainment is generally resource intensive [16, 35] and therefore not often available for monitoring and feedback. A methodological question germane to implementation science is whether the process of implementing a given intervention can be mathematically characterized using low cost or unobtrusive measurement methods [41], what we call of social system informatics (Gallo, CG, Berkel, C, Mauricio, A, Sandler, I, Smith, JD, Villamar, JA, Brown, CH, Implementation Methodology from a Systems-Level Perspective: An Illustration of Redesigning the New Beginnings Program, in preparation) and whether these results can be used to assist implementation decision making processes, potentially reducing human bias and error while reducing the costs associated with scale up and sustainment. This automated alternative to a coding by an expert of the implementation process could use readily available text information, such as an organization’s meeting notes, grantee reports to funding agencies, or email implementation agents, and transcripts of intervention agents, and the target population as programs are delivered. Such text is one type information that we have found useful in identifying key activities and milestones indicative of implementation stage; however, the automated classification of such information has only recently been tested in limited circumstances [16]. In this paper, we present a proof of concept for automating the determination of the stage of implementation using a machine learning algorithm with brief text collected by an implementation broker or intermediary as an evidence-based program is implemented across 40 counties. This automated approach to determining when different stages are entered or exited could pave the way for improved implementation decision-making as programs are scaled up.

Implementation is a process that runs in stages. The number of stages, what each stage represents, and their elements are largely defined by the developer of a specific intervention or specified by the implementation framework that guides the research. Stages are useful for understanding and organizing the tasks and activities that occur earlier versus later in the implementation process, [29] and the level and type of involvement required of each service agency [39]. For example, Aarons and colleagues [1] describe a four-stage framework for the implementation of interventions in the public service sector. These stages are as follows: exploration, adoption decision/preparation, active implementation, and sustainment (referred to as the EPIS framework). With almost any implementation effort, there exists the possibility of multiple stages overlapping or occurring concurrently. This can be very beneficial; for example, monitoring progress on preliminary steps towards sustainment may be useful in predicting whether a health delivery system eventually attains this stage. Many intervention developers have described the implementation process and the scale up of their specific intervention in considerable detail using their own systems, for implementing selected parenting programs that focus on prevention [10, 15, 16, 18–21, 25, 34, 36]. In this particular study, we use the stages of implementation completion (SIC) [7], which involves eight stages, in a randomized implementation trial involving 40 counties in California where multidimensional treatment foster care is implemented [3, 8–10, 23, 30, 32, 40]. We selected this CAL-40 project for proof of concept because of the high quality and strength of the SIC data and use this for development and illustration of the method.

Creating an automatic classifier tools work only requires three things. First, each “message” or a sequence of words pertaining to the implementation process should be assignable by an expert to one or more stages. Some of these assignments need to be classified with certainty, but other messages can and are classified as uncertain by an expert. Further, only a portion of the existing messages need to be coded by an expert into stages. Second, a series of text messages—in our case communication log notes of an intermediary [13]—need to be made available in digital form along with dates of occurrence. Third, an algorithm for text mining the non-classified text needs to be created. In our approach, we use a semi-supervised learning algorithm (described below) to classify what stage one is in based on an ongoing set of communication log notes taken by an intervention broker. The classifier is built on the expert’s classification of messages and applied to unclassified messages to provide probabilities of stage membership. These can then be used to identify when entries, exits, and potential re-entries into these stages occur.

This paper is organized as follows. In the next section, we provide a brief context of our motivating implementation study, what data are available, a high-level overview of the construction of an algorithm for classification, and how it can be used in practice. We also describe preparatory steps including what we term data scrubbing to remove person and place identifiers. This is followed by a more detailed section on keyword labeling, feature extraction, and a description of the novel semi-supervised non-negative matrix factorization algorithm. This section is for readers requiring computational details, but other readers can glean the general approach from the previous section and can skip the details. Next, we illustrate the use of these procedures on text obtained from the CAL-40 project, showing the behavior of this automated classifier based on short sequences of text and how well it can infer the stage and date entrances and exits longitudinally. We end with a conclusion section.

## Methods

### Cal-40 study

For this proof of concept, we utilized the communication log notes from a large-scale randomized implementation trial of multidimensional treatment foster care (MTFC) to examine the efficacy of the proposed text mining approach. A total of 51 counties, 40 from California and 11 from Ohio, were recruited and randomized to the individualized (IND) or community development teams (CDT) implementation model. California counties, whose data are the focus of this paper, were recruited starting May 2006, with follow-up data collected through April 2012. The CDT counties were connected in a learning collaborative model to facilitate knowledge transfer, while counties assigned to IND implemented MTFC under usual conditions (e.g., without social learning and network development across counties). Randomization, allocation concealment, and procedures to minimize contamination across conditions are detailed elsewhere [7, 8].

### Stages of implementation completion

The stages of implementation completion (SIC) measure [7, 29] was developed to provide qualitative and quantitative assessment of the time to achievement of a series of implementation stages. The initial development of the SIC occurred in the context of the CAL-40 randomized implementation trial [7] and has been generalized to many other settings [29]. In brief, the SIC is an observational measure of eight stages used to assess the adoption and delivery of the intervention protocol and the proportion and quality of activity completion within a series of defined implementation stages. The SIC can produce time-based milestone metrics, such as the highest stage achieved and time to stage achievement, along with relevant intervention specific outcomes (e.g., the number of families served). This measure was equally appropriate for counties in the IND and CDT conditions. Research staff collected dates relevant to the events and milestones outlined in the SIC in an ongoing manner throughout the study, thus providing a complete tracking of which stages a county was in at each time [7, 29, 31].

### Communication logs collected by implementation brokers

During the trial, MTFC implementation brokers interacted by phone, email, and in person with the county service organizations to implement this new system of foster care assignments for at-risk children. All interactions were recorded and dated by the intervention brokers through digitized communication log notes they maintained throughout the trial. These notes identified the county and what was the intent or what happened at each contact. Each occurrence generated a single or a few sentences and often included phrases rather than complete sentences. No instructions were provided to the implementation brokers on how detailed these notes should be, nor was there an expectation that these notes would be used later for this automatic classifier.

### Communication log processing and scrubbing

Our text mining would have no generality if it included names of individual people or places in the communication log notes. Thus, our first step was to replace all names of persons and places in the text with placeholders. A second reason for replacing these names with placeholders was to deidentify these records, a requirement of the Oregon Social Learning Center IRB to which the parent research project reported. We used pattern matching computational methods to locate identifiers from the log data. To scrub names from the communication log, we first developed a program that sorted all individual words in the text and matched them against a list of first and last names of team members and employees in each county who were engaged in this project. Matching names were replaced with a unique identifier token (e.g., “person541”) at each location it appeared in the text. Telephone numbers and email addresses were recognized and replaced in a similar fashion. We resolved a few uncertainties using upper and lower case, e.g., the word “bill” could be a person or an invoice, but “Bill” in middle of a sentence was treated as a person. This scrubbing allowed for fast, cheap, and accurate data that maintained the richness of the interactions and protected the identities of the intervention brokers and the county service organizations. Similar technology has been used before to facilitate the sharing of sensitive data between academic institutions [27, 38, 42].

### Processing of individual log notes to formation of longitudinal state trajectories

**Fig. 1:**
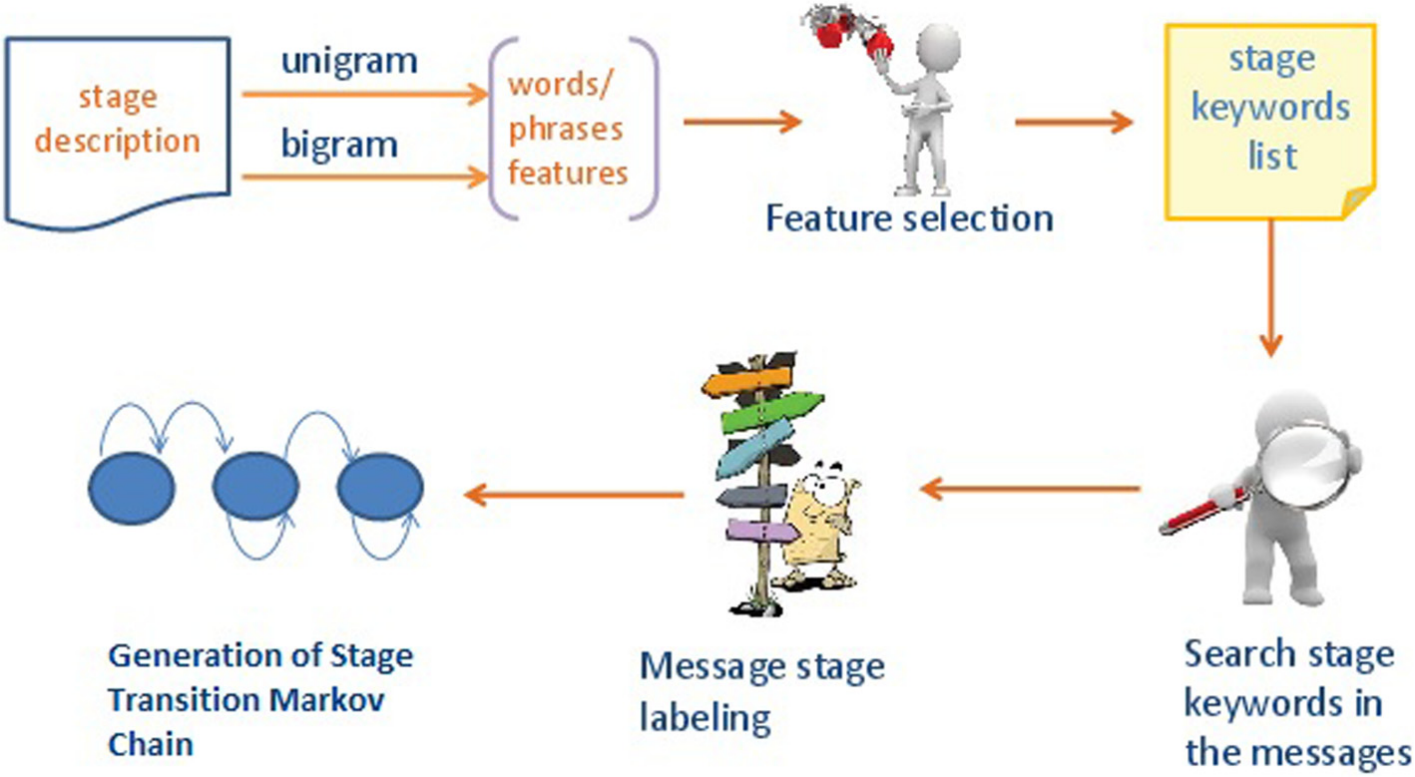
Overview of developing the classification system for summarizing implementation stage transitions

Figure 1 gives a broad overview of the steps used to identify keywords and phrases to build the automated classifier, provide probabilistic labeling of log notes, and produce summaries of each site’s stage transitions across time. Using text mining, we identify potentially important single keywords or unigrams, as well as two-word phrases, or bigrams, and potentially more complex features whose presence in an entry in a communication log is associated with an implementation stage. From here on out, we refer to an entry in a communication log by the term “log note” or with a more general term “message.” A computational procedure is used to determine an optimal set of these keywords, a step known as feature selection. Then, each log note is searched for these selected features and labeled into stages according to its probability assessment. The final step is to construct summary representations of these using Markov or other more complex modeling approaches.

### Identification of keywords from the text and formation of the classification rule

**Fig. 2:**
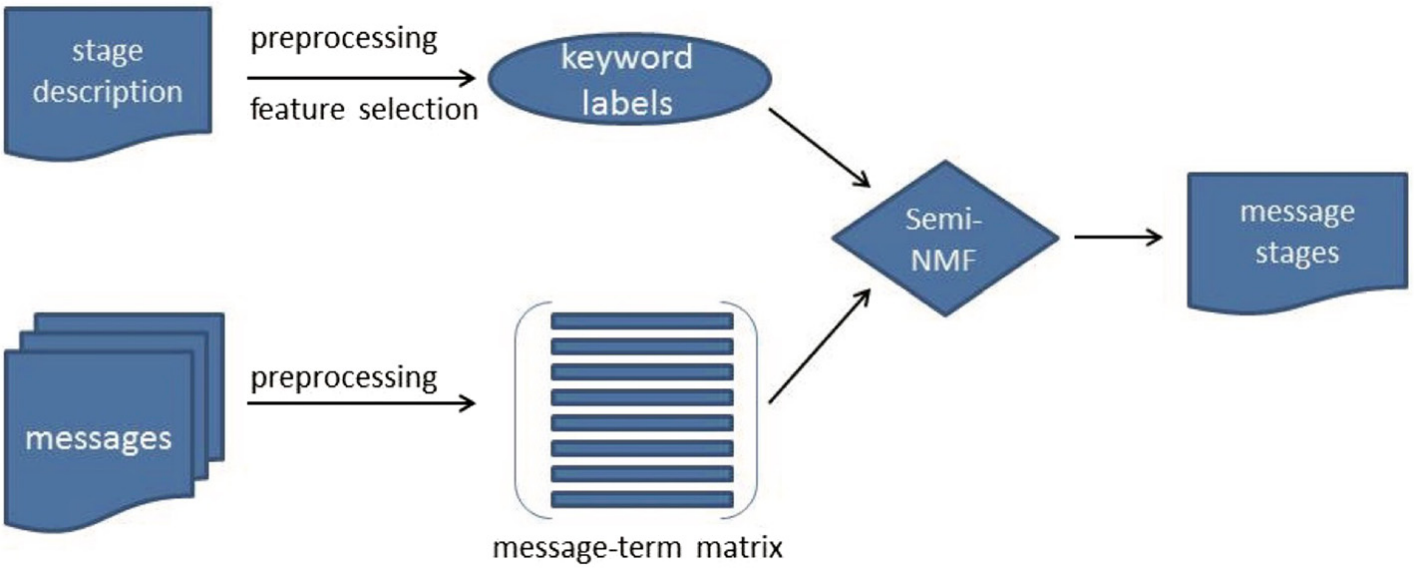
Schematic of the classification rule

The key methodological challenge is to identify which words and phrases should be identified from a log note. This can be accomplished through steps illustrated in Fig. 2. Our text mining involves two sets of information. First, from the description of each stage, keywords pertaining uniquely to that stage are identified. Second, other potential unigrams or bigrams are identified based on log notes that are previously classified by experts as well as their frequency of their appearance. In the second row of this figure, we count the occurrence of each of these keywords in each log note. A machine learning algorithm—a novel version of a semi-supervised non-negative matrix factorization (semi-NMF), which has shown superior performance to other alternatives—is used to determine probably stages for each log note.

The stage prediction problem considered in this paper has two important characteristics. First, the same log note may appear in multiple stages. Thus, instead of classifying each log note into a unique stage, we try to assign with a probability value the log note to each stage, where the probability values of the log note across the stages sum to 1. Second, log notes are classified independently across time and site. This temporal feature makes the stage assignment problem a natural fit for incremental classification; here, a new log note is processed for the probabilistic class assignment without referring to the probability values calculated for the previous log notes from the site.

## Details on machine learning

Two sets of data are available as input for the training set. The first data set, *D*_stage_, consists of the stage descriptions in text obtained from the published SIC measure, organized as follows: let 1,…,*S* be the stages of the process. Then, *D*_stage_ is {*A*_*i*_∣*i*,1≤*i*≤*S*}, where for each *A*_*i*_, *i*, 1≤*i*≤*S*, is a set of phrases/sentences that describe activities and events to occur in the stage *i*, e.g., “first study-initiated contact for pre-implementation” and “first county response to first contact for pre-implementation planning”. The second data set, *D*_log_, is the collection of log notes with known classification into stages based on a human expert. This matrix is organized as follows: let *L* be the number of distinct adoption efforts (i.e., the number of distinct counties in which the brokers made an attempt for adoption). Then, *D*_log_={*B*_*i*_∣1≤*i*≤*L*}, where for each *i*, 1≤*i*≤*L*, *B*_*i*_ comprises the time-stamped log notes from the *i*th adoption effort. For each *i*, 1≤*i*≤*L*, *B*_*i*_ is a series of pairs $\{ (t_{ij}, m_{ij}) \}_{j=1}^{T_{i}}$, where *T*_*i*_ is the total number of log entries classified in stage *i* and for each *j*, 1≤*j*≤*T*_*i*_, *t*_*ij*_ is the date and time the activity/event took place and *m*_*ij*_ its text, where $t_{i1} < \cdots < t_{i T_{i}}\phantom {\dot {i}\!}$.

The two data sets are processed in order, first *D*_stage_ and then *D*_log_. The stage data set *D*_stage_ is processed for keyword selection. This is a process for selecting a set of “words” that are likely to occur in stage descriptions of only one stage. Since a word can take different forms (e.g., a verb and its third person singular form or its gerund), the descriptions are first processed for “stemming”—stripping each “word” to its stem (e.g., “tak-” is the stem for “take,” “taking,” “took,” and so on). For this purpose, the Porter stemmer [28] method is used. After stemming, the stems with the aforementioned discriminating power are selected using feature selection techniques [24] (see Section Keyword labeling via feature selection for details). Based on the descriptions and the keywords, one may be tempted to generate a keyword-to-stage mapping matrix, whose *ij* entry represents the importance of the *i*th keyword in the log notes for the stage *j*. However, noting that those who generate the descriptions are unlikely to be those who generate the log notes, the use of such a matrix may result in inaccurate prediction of stages. Rather, the proposed method calculates this importance through training.

The training set of log notes, *D*_log_, is processed with keywords extracted as auxiliary input after scrubbing as described above. Let *K* be the number of keywords thus extracted and let *W*={*w*_1_,…,*w*_*K*_} be the keywords. Each log note in *D*_log_ is processed for stemming as in the same manner as in *D*_stage_ and then the occurrence of each keyword is counted. This produces a keyword-count vector of dimension *K*. After normalizing the entries by dividing the total count, each count vector is reduced to a real vector of dimension *K* in which the entries are non-negative and the total of the entries sum to 1. By assembling these *K*-dimensional normalized keyword frequency vectors as rows, an *L*×*K* non-negative matrix *X*=(*x*_*ij*_) is obtained, in which *x*_*ij*_ is the entry corresponding to the *j*th keyword in the *i*th log note. Next, the keywords *W* and the occurrence matrix *X* are processed with non-negative matrix factorization for the purpose of factorizing each row of *X* as a linear combination of the rows of a stage-keyword matrix with non-negative coefficients. In an ideal situation, the descriptions are sufficient to characterize distinctions among the stages and so the keywords chosen are sufficient for computationally distinguishing among the stages of the log notes with no additional human input. Unfortunately, this is not the case, and so an assumption is made that a small fraction of the log notes are already labeled with their corresponding stage IDs by a domain expert and the decomposition of the log note vectors is carried out using this additional piece of information as a guide in a “semi-supervised” manner, which will be described in detail in the next section.

### Keyword labeling via feature selection

Once the description-term representation has been obtained, keyword selection is made using the max-relevance and min-redundancy framework as presented in [24]. Here, the keywords are selected in sequence, one after the other, until the number of selected keywords has reached a target number. At each step, a new keyword is chosen so as to (a) minimize the resemblance of the new keyword to the keywords chosen in the previous steps and (b) maximize the representation of the documents in the collection when any other other than the previously chosen keyword and the new keyword are removed. The idea can be formulated as an optimization problem as follows:

Suppose that the task is to select a set of keywords in sequence for stage *i*. Let *A*_*i*_ be the stage descriptions for stage *i* and let *P*_*i*_ be the set of all unique words appearing in *A*_*i*_ (after stemming). Let *k*≥1 and suppose that we have already selected *k*−1 keywords. Let *W*_*k*−1_ be the set of these *k*−1 keywords. The *k*th keyword shall be selected from the remaining candidates for keywords; i.e., *P*_*i*_−*W*_*k*−1_. The selection is made using the following formula from [24] that attempts to minimize the redundancy and to maximize concordance:

1 $$  \max_{w_{q}\in P_{i}-W_{k-1}}\left(\text{sim}(w_{q};P_{i}) -\frac{1}{k-1}\sum\limits_{w_{p}\in W_{k-1}}\text{sim}(w_{q};w_{p})\right).  $$

Here sim is the similarity that utilizes the cosine similarity function. The function sim utilizes the cosine similarity function. The cosine similarity of for two *r*-dimensional vectors (*x*_1_,…,*x*_*r*_) and (*y*_1_,…,*y*_*r*_) by

$$\frac{x_{1}y_{1} + \cdots + x_{r} y_{r}}{\sqrt{{x_{1}^{2}} + \cdots + {x_{r}^{2}}}\sqrt{{y_{1}^{2}} + \cdots + {y_{r}^{2}}}}. $$

For similarity, the first entry sim(*w*_*q*_;*P*_*i*_) compares the vector consisting of the number of occurrences of *w*_*q*_ in the documents of *A*_*i*_ and the vector consisting of the total number of words in the documents of *A*_*i*_ (if *A*_*i*_ has *m* documents, both are *m*-dimensional). The second entry compares such vectors for *w*_*p*_ and for *w*_*q*_.

The computational complexity of this incremental selection is *O*(*k*∥*P*_*i*_∥).

### Semi-supervised non-negative matrix factorization

In the semi-supervised learning model employed, it is assumed that a fraction of the log notes and a fraction of the keywords are labeled, each with its correct stage number. The label of a log note comes from a domain expert who is able to assert that it belongs to a unique stage. The label of a keyword comes from the feature extraction process; that is, each keyword that uniquely appears in a single stage is designated as the labeled keywords, with the number of the stage in which they uniquely appear being the stage label. While the proportion of stage-labeled log notes is a feature determined by our design, the proportion of the other is a parameter that can be determined based on the choice of the keywords. That is, if there are no such uniquely occurring keywords, the proportion is necessarily 0. Of course, not all the keywords with unique association with a single stage should be used for this purpose, since those who provide the descriptions can be different from those who leave the log notes. Thus, the uniquely occurring keywords may be used for multiple stages by the log note takers.

The non-negative matrix factorization here computes two non-negative matrices: *F* of dimension *L*×*S* and *G* of dimension *K*×*S*. Each row of *F* sums to 1 and for each *j*, 1≤*j*≤*S*, the *j*th entry of the row represents the calculated probability that the log note corresponding to the row belongs to stage *j*. Each row of *G* sums to 1 and for each *j*, 1≤*j*≤*S*, the *j*th entry of the row represents the probability that the keyword corresponding to the row belongs to stage *j*.

Let *F*_0_ be the *L*×*S* matrix that represents the partially assigned labels of the log notes. That is, for each *i*, 1≤*i*≤*L*, if the *i*th log note is not part of the labeled data, the *i*th row of *F*_0_ is all 0, and if the *i*th log note is part of the labeled data and is labeled as stage *j*, 1≤*j*≤*S*, the *i*th row of *F*_0_ is a vector in which the *j*th entry is 1 and all the other entries are 0. Let *G*_0_ be the *K*×*S* matrix similarly defined for the keywords. That is, for each *i*, 1≤*i*≤*K*, if the *i*th keyword is not part of the labeled data, the *i*th row of *G*_0_ is all 0, and if the *i*th keyword is uniquely associated with stage *j*, 1≤*j*≤*S*, the row is all 0 except for the *j*th entry which is 1. Let *C*_0_ be the *L*×*L* diagonal matrix in which the *i*th diagonal entry is 1 if the *i*th log note is part of the labeled data and 0 otherwise. Similarly, let *C*_1_ be the *K*×*K* diagonal matrix in which the *i*th diagonal entry is 1 if the *i*th keyword is part of the labeled data and 0 otherwise.

The semi-supervised non-negative matrix factorization to be done can be formulated as the following optimization problem:

2 $$\begin{array}{@{}rcl@{}} \min_{F, H, G} &&\left\{ \| X - F H G^{T} \|^{2}\right.  \\ && + \alpha \cdot \text{trace}\left((F - F_{0})^{T} C_{0} (F - F_{0})\right)  \\ && +\left. \beta \cdot \text{trace}\left((G - G_{0})^{T} C_{1} (G - G_{0})\right)\right\}, \end{array} $$

where *F* is *L*×*S*, *G* is *K*×, and *H* is *S*×*S* are nonnegative real matrices.

Also, *α*>0 and *β*>0 are parameters, which are used to define the extent to which *F*≈*F*_0_ and *G*≈*G*_0_ are enforced, and *H* is an *S*×*S* matrix that represents correlations among the stages. In general, involving these parameters make the model more generic and it allows certain flexibility. For example, in some cases, if the manually labels are not very accurate or ambiguous, we can set a smaller *α* or *β* so that the final results are not dependent on *F*_0_ or *G*_0_ too much. In our experiments, since the human labeler is very confident with the labels, we set *α* and *β* to be 1. The solution of the above optimization problem, the computational algorithm, and the proof the convergence of the proposed algorithm are listed in the Appendix.

## Results

Here, we present a brief description of the input data and findings of this method as applied to the CAL-40 study.

### Data description and preprocessing

**Summary descriptions of CAL-40 data.** The data set consists of 4589 log notes from 40 California counties. Each log note is labeled by a project coordinator with a stage number, ranging from 1 to 8 as follows:

- Stage 1—Agreement to consider implementation
- Stage 2—Pre-implementation
- Stage 3—Recruitment plan and review
- Stage 4—Training schedule
- Stage 5—Developer/administrator call
- Stage 6—Clinical meeting
- Stage 7—Implementation review
- Stage 8—MTFC symposium and certification application

Of the 40 counties, 31 did not go beyond stage 3. The remaining 9 counties proceeded beyond stage 3. In addition to the 4589 log notes, we also used 76 stage description sentences which were incorporated into the log notes and used as the labeled data. Table 1 shows the detailed data distribution for labeled and unlabeled data. In the stage prediction experiments, we use four categories representing stages 1, 2, 3, and 4 or above.

**Table 1:** Data distribution

	Stage 1	Stage 2	Stage 3	Stage 4 or later
Unlabeled	781	2937	183	688
Labeled	8	9	12	47

As described in Section Keyword labeling via feature selection, a pre-designated number of keywords were selected to be used as labeled words. The number of keywords was chosen experimentally: we used a different number of keywords in the experiments and selected the solution producing the best results. Some typical keywords were “feasibility,” “pre-implementation,” “referral,” “initiate,” “certification,” etc.

### Prediction accuracy

We use accuracy as the evaluation metric to compare the prediction performance of our non-negative matrix factorization-based classifier with other supervised learners such as SVM and Naïve Bayes classifier. Accuracy was defined based on the proportion of correct classifications of the log notes.

$$\text{Accuracy} = \frac{\text{Number\_of\_correct\_predictions}} {\text{Total\_number\_of\_log\_notes}} $$

#### Number of keywords

**Fig. 3:**
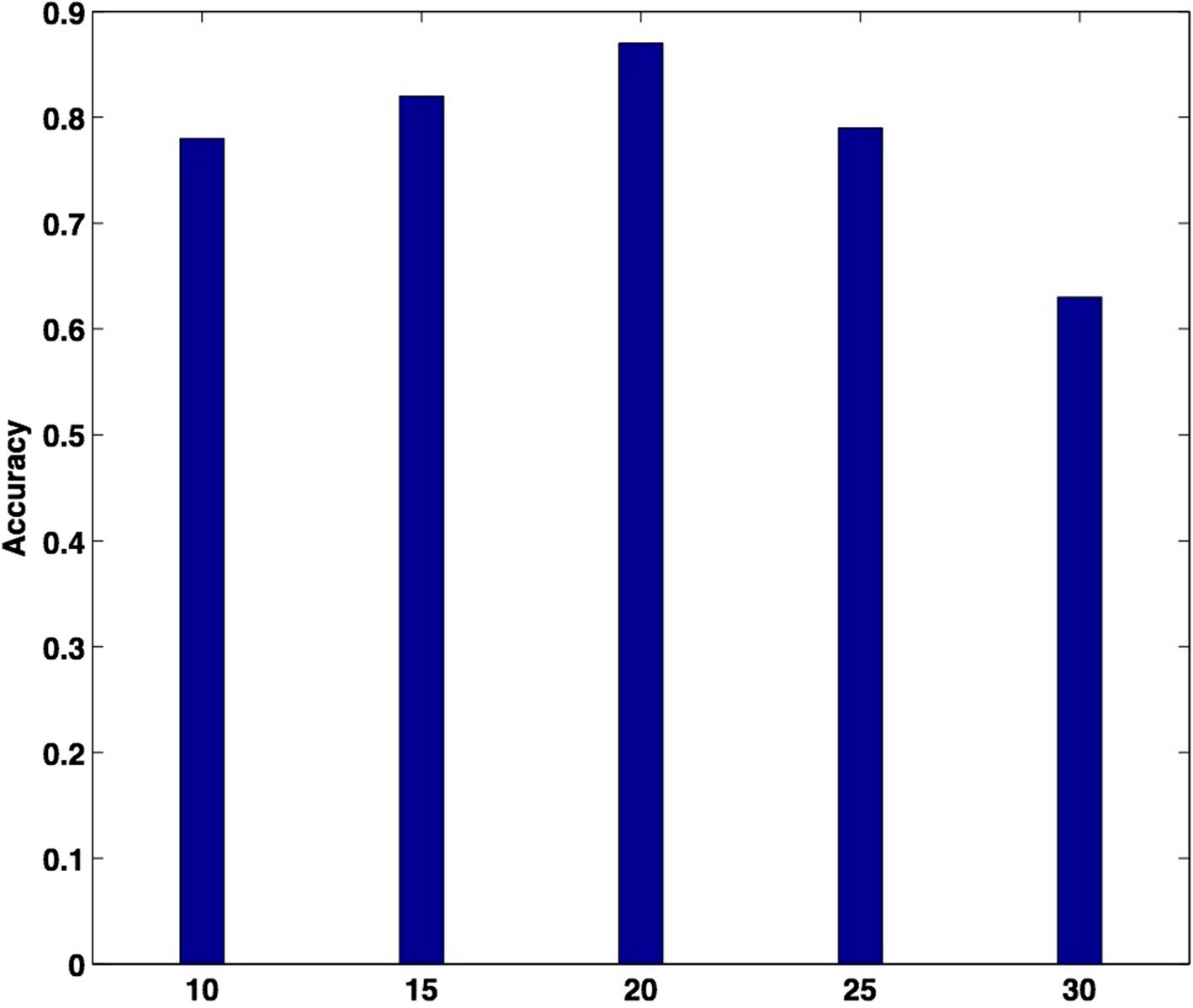
Accuracy of the semi-NMF-based classifier with respect to different numbers of keywords

First, we compared the prediction accuracies using different numbers of keywords. From Fig. 3, we can see that 20 keywords produced the best prediction accuracy. Clearly, if the number of keywords is too small, there is not enough information carried by the keywords and so the accuracies are low. Interestingly, it is not the case that the more keywords, the better results. The reason that the accuracy peaks at 20 and then declines beyond 20 is perhaps that low-ranked keywords have the effect of confusing the classifiers.

#### Comparison of classification accuracy

For comparison, two alternative machine learning methods are compared to the method we described in the previous section.

- Naïve Bayes: a Naïve Bayes classifier is a scalable and effective supervised learner.
- Support vector machines (SVM): SVM is one of the most widely used supervised learning model, and it is robust and effective to solve data classification problems [11]. In experiments, we use linear kernel which has been shown effective in text mining tasks.
- Semi-NMF: this does not reply on prior keyword knowledge. This uses partial log note labels in the aforementioned algorithm.

**Fig. 4:**
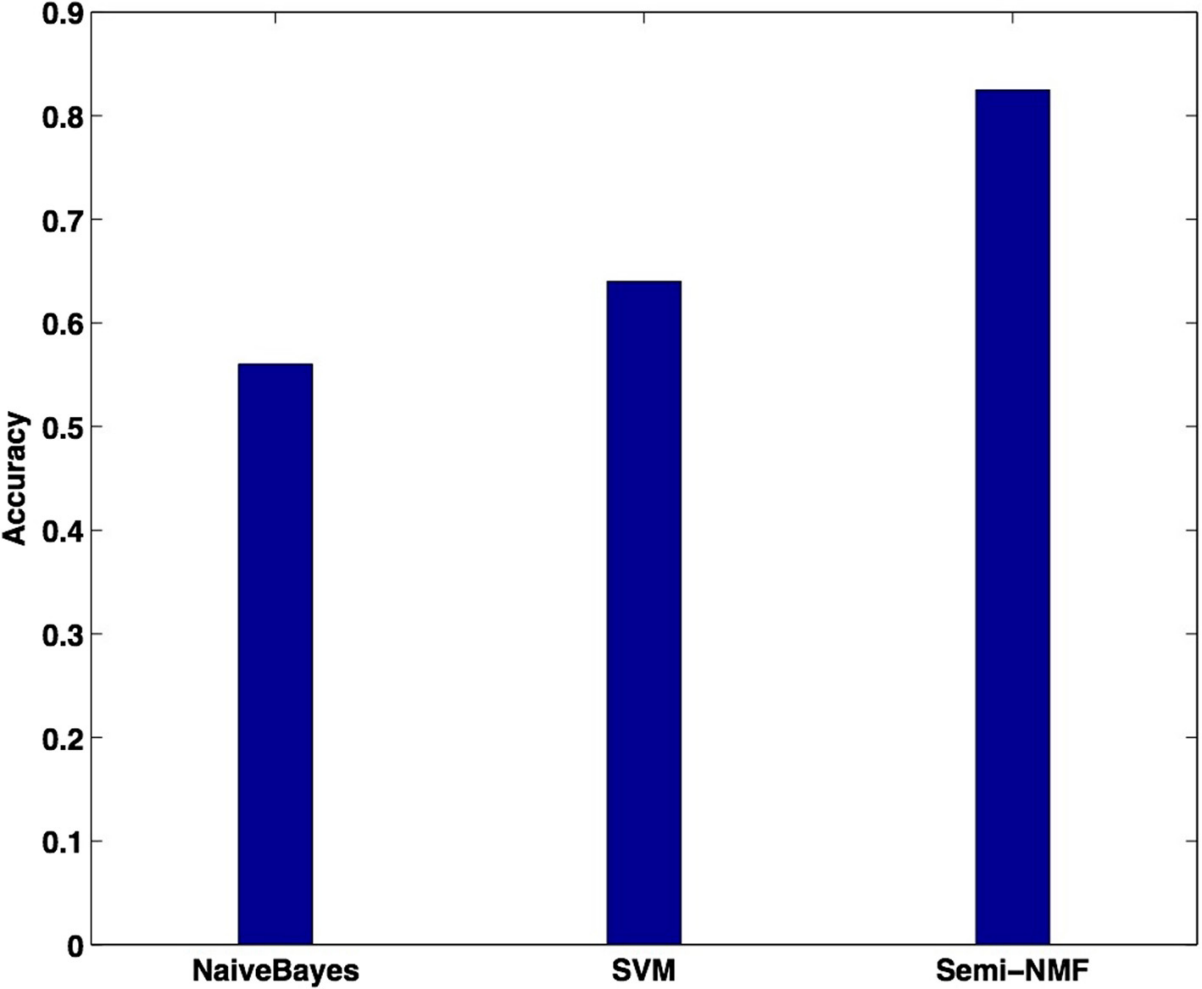
Prediction accuracy comparison with different methods

Figure 4 compares the accuracy of these three methods. We observe that the semi-NMF with prior keyword knowledge model can produce 82.5 % in accuracy. It clearly outperforms SVM and Naïve Bayes classifiers because semi-NMF is a semi-supervised learner. The advantage of the method may also be in that it makes use of both labeled and unlabeled data for training a better model to predict the unknown labels.

### Finding start and end dates of each stage

**Fig. 5:**
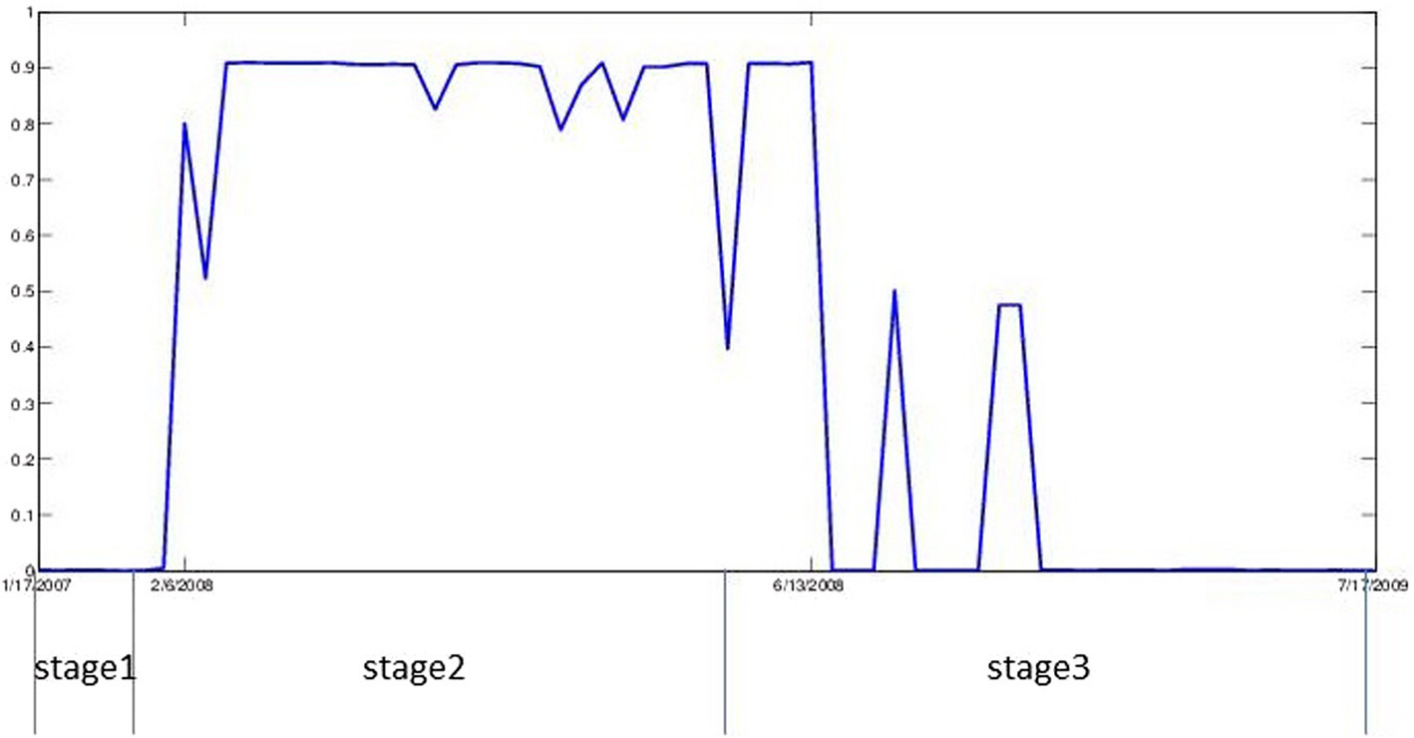
Example start and end date discovery on county 17 stage 2 data

**Fig. 6:**
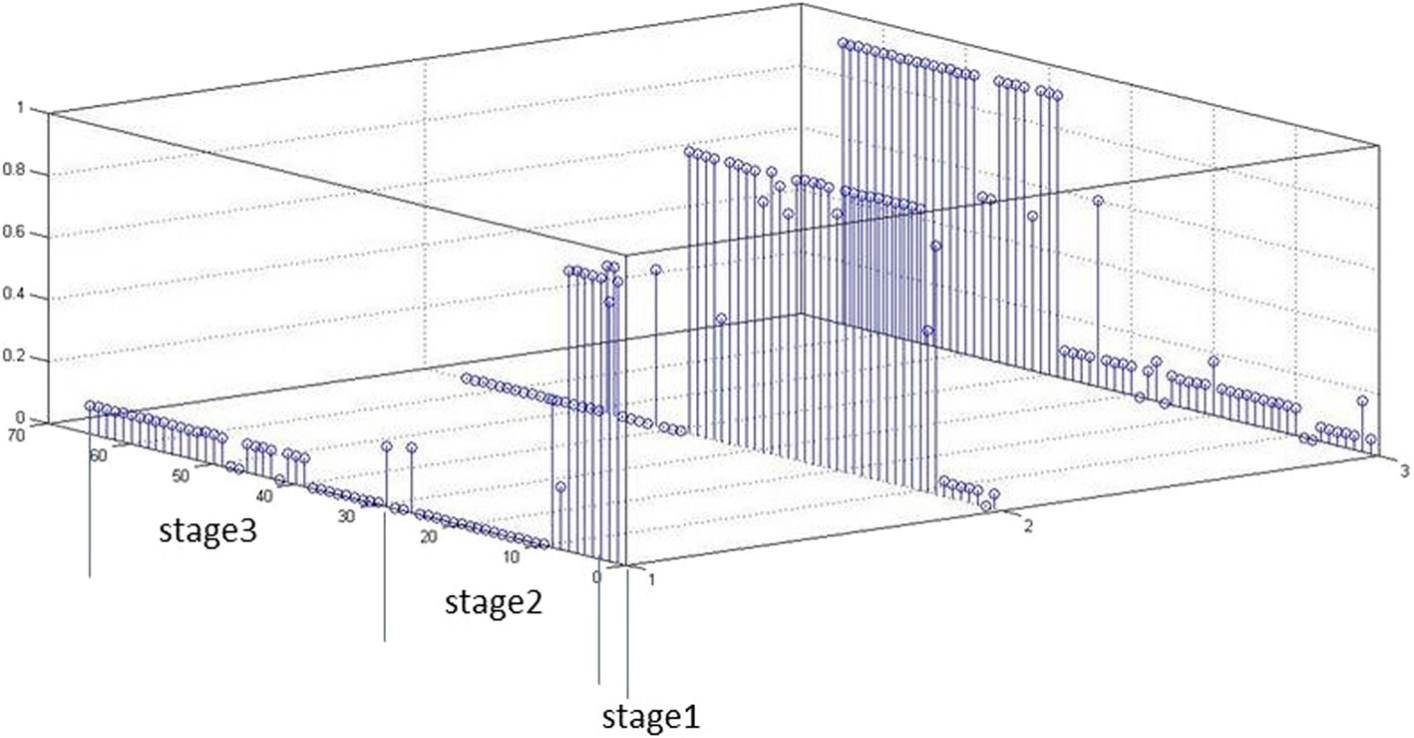
Transitions of county 17 through stages 1 to 3

Since semi-NMF makes probabilistic assignments of log notes to stages, we can infer the start and end dates of a stage by detecting the change in the probabilities. Figure 5 shows the changes in the probability value of log notes being in stage 2 in the County 17 data. From the figure, we see that the probability suddenly increases on 6 February 2008 and suddently decreases on 13 June 2008. It may be inferred from that County 17 may have be in stage 2 between 6 February 2008 and 13 June 13 2008. Comparing this to the human-labeled ground-truth (which is shown below the time axis in the figure), we find that 55 out of 65 log notes are correctly classified. Figure 6 shows the entire picture of the county 17 prediction.

As mentioned earlier, only nine counties arrived at stage 4 in the original implementation trial. By checking the predicted stage label, we found that 7 of the 31 counties that had yet to reach stage 4 contained a log for which our classifier asserted at least one log note was classified in stage 4. This results in an error rate of 7/31=22.6 *%* for stage 4 classification.

### Transitions between states over time

To represent how changes occur over time in a single county, one can imagine generating Markov chain models for state transitions relying upon stage classifiers.

Table 2 shows the confusion matrix generated using 120 samples covering stages 1–4. Overall accuracy is 83.3 %. While the accuracy in stages 3 and 4 appear lower than the other two, the performance on the first two appear high.

**Table 2:** Confusion matrix

Classification results	True labels			
	1	2	3	4
1	*29*	1	4	2
2	1	*63*	5	0
3	1	2	*5*	1
4	0	0	3	*3*

**Fig. 7:**
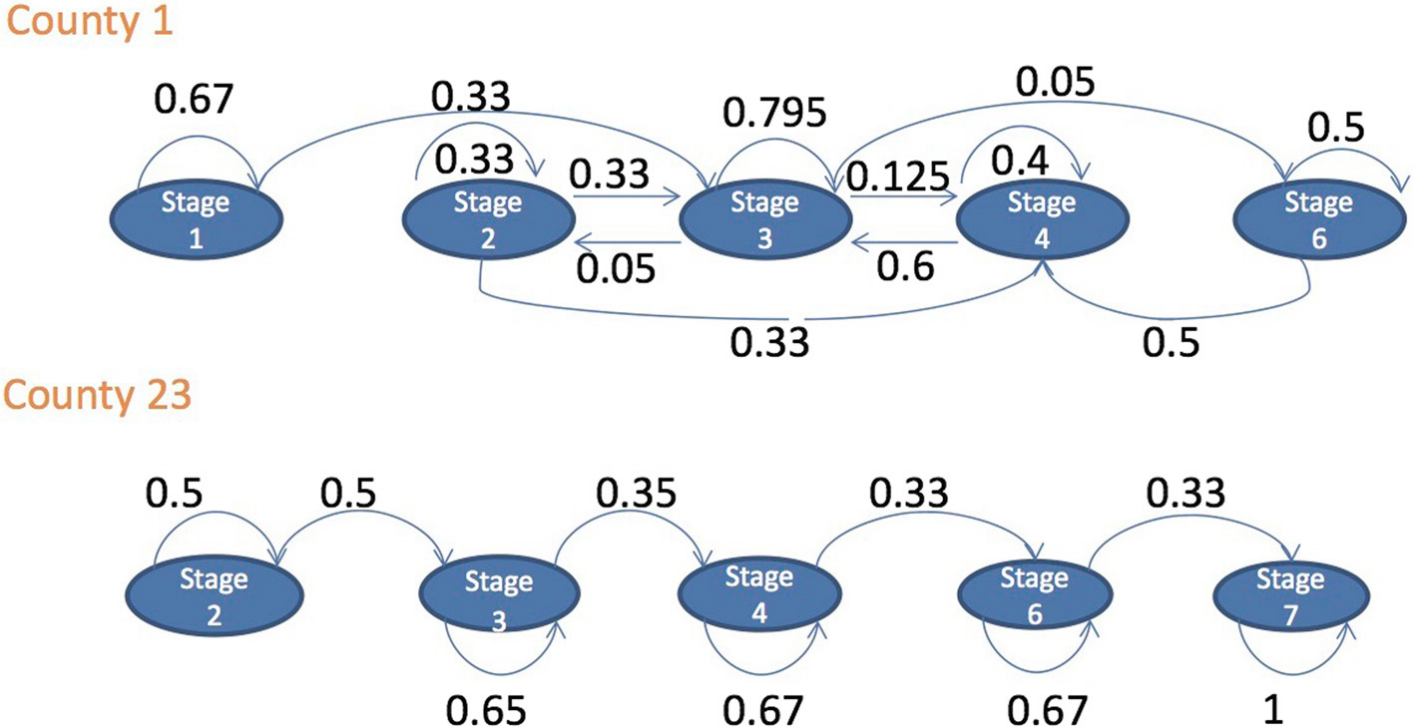
Transitions between stages for two counties using Markov modeling

Ideally, these models produce estimates of remaining in a state or transitioning to another. Figure 7 shows widely different patterns of transition for two counties. County 1 is more likely than County 23 to remain in stage 1, based on its higher probability of 0.67 of remaining in stage 1 compared to that of County 23.

## Discussion

For this proof of concept, we developed a computational approach for monitoring transitions of a health delivery system through identified stages of implementation. It relies on text information that can be collected readily and used to provide feedback at appropriate levels from front line clinicians and facilitators to agency managers and to funding agencies. A novel semi-supervised machine learning algorithm was developed that used as input two types of information; the first involved text descriptions of each stage and the second consisted of log notes from an intermediary in which some were coded by a human expert. We provided brief illustrations of how such information could be used to provide summaries of implementation progress involving single sites or comparisons of sites. Quantitatively, the accuracy of these automated classifications has reached acceptable levels for a range of uses that we explored in the results section.

We recognize that some uses would likely require more accurate classifications. In particular, our over-projection of counties to have reached stage 4 suggests that in real applications, there would need to be a tuning of the algorithm that takes into account to relative costs of omitted and committed classification errors. There are many possibilities for improving accuracy that we have not investigated yet. For example, our classifications of each log note are all done independently; combining the classifications across a proximal series of such notes can provide improved decision making, as we have demonstrated in another automated implementation project (Li, Y, Gallo, CG, Mehrotra, S, Liu, L, Brown, CH, Recognizing emotion in speech for behavioral intervention studies, submitted). Furthermore, we have developed a general concept for using a long series of unobtrusive or lower quality measures involving complex human interactions and behaviors in place of highly accurate but costly measures observed by humans. These methodological approaches fall under the name of social systems informatics and can readily outperform human coding when large amounts of automatically coded data are available (Gallo, CG, Berkel, C, Mauricio, A, Sandler, I, Smith, JD, Villamar, JA, Brown, CH, Implementation Methodology from a Systems-Level Perspective: An Illustration of Redesigning the New Beginnings Program, in preparation).

We note several potential uses of this method. One challenge implementation scientists face is the successful scale up of effective interventions. The comparatively high costs of human monitoring of implementation necessarily increase proportional to the number of sites involved when scaling up. But the costs of using a computational algorithm for automatic classification are virtually the same regardless of the number of sites, and the availability of additional log notes pertaining these sites should lead to improved classifications at a local level and more generalizable knowledge as well. Monitoring of supervision and program fidelity could be aided by the methodology described here. In particular, a facilitator’s utterances can be transcribed computationally to text using existing technology and used as input to an automated classification system [14]. Computational models characterizing the implementation process can be used as a guide for newly hired intervention delivery agents and serve to “alert” them to potential problems (i.e., drift) as defined by the intervention developer. A third challenging problem for implementation is to monitor and assist sites in sustaining the delivery of effective evidence-based programs especially after federal or local funding ends. Indeed, many community-based organizations that are funded by local, tribal, state, or federal agencies are provided little funding for infrastructure and therefore have limited capacity to detect or anticipate problems with the implementation process or plan for long-term sustainment. Building a monitoring system that uses as input text from required reports of these organizations can be an ideal way to monitor steps they are taking (or not taking) towards sustainment throughout the grant period. Finally, such tools could be used as a first stage measure in randomized implementation trials [4, 6, 8, 22, 26, 37], backed up by a stratified sampling approach that adds more accurate human coding for large implementation evaluations.

This automated system is an example of a broader set of computational technologies that we believe could provide important supports for successful implementation. Brown and colleagues [5] describe a general model that identifies eight areas where computational and systems science methods can aid the scale up, dissemination, and sustainability of an effective program. These eight areas represent important points of entry where a technology, such as the proof of concept presented here. The computational methods described by Gallo and colleagues [17] and Atkins and colleagues [2] can support the implementation efforts of a service delivery agency. We note that Gallo and colleagues used computational linguistics and natural language programming to detect the component of “joining” from digitally recorded sessions of the Familias Unidas program. The automatic detection of this key dimension of fidelity means that it is possible to shorten the time between when a session happens and the feedback available for that session. Atkins and colleagues used computational linguistic methods to evaluate fidelity to motivational interviewing for a similar purpose.

Such a system could be scaled up with minimal effort and cost in several ways. First, that mimics the stage identification in an implementation process typically rated observationally by a trained expert using the SIC. We make a bold assumption that to a large degree, the stage of implementation is represented in the words exchanged between the intervention developer and the intervention adopters and that pertinent information in these written exchanges can be recognized by an algorithm. The present study tests this assumption. The results of this proof of concept study support our assertion that the adoption and implementation process of an effective program can be supported through the use of computational science methods. We used a computational method where the communication log notes from a randomized implementation trial of MTFC were used to successfully distinguish between implementation in the first three stages and beyond stage 4 of the SIC measure.

The work presented in this study is not intended to replace existing human monitoring of an implementation process. For those systems where expert implementation specialists are monitoring key processes, and those where the SIC measurement system can be maintained, it is likely that they will continue being used effectively. There may be opportunities for blended systems where a first level automated system can focus human attention where needed. We envision computational methods existing synergistically alongside human ratings to increase efficiency and reduce costs by analyzing large amounts of easy to collect data in an ongoing manner. There is also a great benefit in using quality measurements such as the SIC to specify the distinct stages relevant to an implementation strategy. We foresee greater use of such measures as the SIC now that a Universal SIC is being developed [33].

Addressing this challenge is of particular importance for consumers of effective programs, like federal and state service delivery agencies, as well as developers and promoters of effective interventions. Service delivery agencies commit resources in the adoption of an effective intervention expecting improved health outcomes for the people in the communities they serve. This is especially true given the proven success of programs based on previously completed efficacy and effectiveness studies.

There are multiple potential applications of this method in HIV prevention that can be used by public health agencies and community-based health centers. For instance, public health departments who fund implementation of evidence-based prevention interventions across multiple sites often require routine progress reports to monitor implementation and achievement of health outcomes. This method could facilitate the monitoring of implementation and decrease the time to review reports, thus allowing for closer to real-time feedback on program improvements.

Many community-based health centers clinics throughout the country are in the early stages of implementation of pre-exposure prophylaxis (PrEP), a medication with demonstrated impact on preventing HIV infection. Automated monitoring of the progress of implementing PrEP within these community-based organizations as they are monitored by local public health departments could provide one important application of the method provided here. Also, these methods could be applied to individual case notes. For example, computational methods that perform linguistic mining of notes in electronic medical records can also facilitate the monitoring of an individual client’s progression along the PrEP continuum of care, routine clinical follow-up and monitoring, and adherence to PrEP medication. This method could also provide real-time feedback to program implementers of potential threats to adherence that could be prevented with timely information.

Our proof of concept had several limitations. Since our work only involving a single implementation study of MTFC measured by the SIC, we cannot be certain that this procedure will work with other measurement systems. With Saldana and colleagues’ development of a Universal SIC [33], the methods developed here will need to be fine-tuned for application across programs beyond this particular evidence-based program. Secondly, what successes this algorithm has had dependeds on the distinctness of the stages, sufficient consistency in the way that the log notes are produced, and the quality and quantity of human coding of a portion of the log notes. Little information is available to guide others in deciding the magnitude of these important factors.

## Conclusions

The detection of the quality, speed, and milestone attainment of the implementation process of effective programs is of paramount concern for implementation scientists, policy makers, and practitioners [1, 3]. Federal and state agencies that invest in programs of research to develop effective programs, healthcare delivery systems charged with delivering the best available care, and the consumers and beneficiaries of such programs are also highly invested in supporting the adoption and implementation of effective programs. These agencies often do not have the capacity to detect or anticipate problems with the implementation process.

## Appendix

### Computational algorithm

To solve the optimization problem in Eq. 2 the following update rules adapted from [12] can be used.

- Construct an *L*×*S* matrix *F* ^′^ by setting for each *i*, 1≤*i*≤*L*, and for each *j*, 1≤*j*≤*S*,
  3 $$  F'_{ij} \leftarrow F_{ij} \frac{(X G H^{T}+\alpha C_{0} F_{0})_{ij}}{(F F^{T} X G H^{T}+\alpha C_{0}F)_{ij}},  $$
  and then set *F* to *F* ^′^.
- Construct an *S*×*S* matrix *H* ^′^ by setting for each *i*, 1≤*i*≤*S*, and for each *j*, 1≤*j*≤*S*,
  4 $$  H'_{ij} \leftarrow H_{ij} \frac{(F^{T} X G)_{ij}}{(F^{T} F H G^{T} G)_{ij}},  $$
  and then set *H* to *H* ^′^.
- Construct a *K*×*S* matrix *G* ^′^ by setting for each *i*, 1≤*i*≤*K*, and for each *j*, 1≤*j*≤*S*,
  5 $$  G'_{ij} \leftarrow G_{ij} \frac{(X^{T} F H + \beta C_{1} G_{0})_{ij}}{(G G^{T} X^{T} F H + \beta C_{1} G)_{ij}}.  $$
  and then set *G* to *G* ^′^.

The algorithm consists of an iterative procedure using the above update rules until convergence. The detail procedure is shown in Algorithm 1.



### Algorithm correctness and convergence

Updating *F*, *G*, and *H* using the rules above leads to an asymptotic convergence to a local minima. This can be proved using arguments similar to (Ding et al. 2006). We outline the proof of correctness for updating *F* since the squared loss term that involve *F* is a new component in our models.

#### **Theorem**

At convergence, the solution satisfies the Karuch, Kuhn, Tucker optimality condition (KKT condition, for short), i.e., the algorithm converges correctly to a local optimum.

#### **Proof of the Theorem 1**

Following the theory of constrained optimization (Nocedal and Wright, 1999), we minimize the following function

$$\begin{array}{@{}rcl@{}} L(F) &=& {\|X-FHG^{T}\|}^{2}+\alpha \cdot \text{trace}[(F-F_{0})^{T}C_{0}(F-F_{0})] \\ &\,& ~~~~ +\beta \cdot \text{trace}[(G-G_{0})^{T}C_{1}(G-G_{0})] \\ \end{array} $$

Note that the gradient of *L* with respect to *F* is,

6 $$ \frac{\partial L}{\partial F} = -2XGH^{T} + 2FHG^{T}GH^{T} + 2 \alpha C_{1}(F-F_{0}).   $$

and a similar relation holds for *G*.

The KKT complementarity condition for the nonnegativity of *F*_*ik*_ gives

7 $$ [-2XGH^{T} + FHG^{T}GH^{T} + 2 \alpha C_{0}(F-F_{0})]_{ik}F_{ik} = 0.   $$

This is the fixed point relation that local minima for *F* must satisfy. Given an initial guess of *F*, the successive update of *F* using Eq. (3) will converge to a local minima. At convergence, we have

$$ F_{ik} = F_{ik} \frac{(XGH^{T} + \alpha C_{0}F_{0})_{ik}}{(FF^{T}XGH^{T} + \alpha C_{1}F)_{ik}}. $$

which is equivalent to the KKT condition of Eq. (7). The correctness of updating rules for *G* in Eq. (5) and *H* in Eq.(4) have been proved in [12]. Note that we do not enforce exact orthogonality in our updating rules since this often implies less accurate class assignments.

## Abbreviations

CAL-40: California 40-county implementation trial
CDT: Community development teams
IND: Individualized county implementation
IRB: Institutional review board
MTFC: Multidimensional treatment foster care
NMF: Non-negative matrix factorization
SIC: Stages of implementation completion
SVM: Support vector machine
